# Pediatric Herpes Zoster in a 10-Year-Old Boy With Delayed Immunizations: A Case Report

**DOI:** 10.7759/cureus.80787

**Published:** 2025-03-18

**Authors:** Brandon Raquet, Kaitlin N Murphy, Lianna Lawson

**Affiliations:** 1 Internal Medicine, Edward Via College of Osteopathic Medicine, Blacksburg, USA; 2 Family Medicine, Lawson Family Medicine, Danville, USA

**Keywords:** herpes zoster reactivation, herpes zoster virus, pediatric derm, vaccination status, vesicular rash

## Abstract

Herpes zoster (HZ), caused by the reactivation of latent varicella-zoster virus, is a condition typically associated with older adults and immunocompromised individuals, though it can also occur in children. Pediatric HZ is rare but can follow a natural varicella infection, particularly in unvaccinated children. We report a case of a 10-year-old immunocompetent boy with a history of natural varicella infection at two years old and a delayed vaccination schedule who developed HZ following a viral illness. He presented with a painful, pruritic vesicular rash in a T4-5 dermatomal distribution, and his symptoms resolved with symptomatic management. This case underscores the critical role of routine varicella vaccination in preventing primary varicella infections and reducing the risk of HZ. While pediatric HZ is often mild, clinicians should remain vigilant for its occurrence and potential complications, particularly in unvaccinated children or those with incomplete immunization histories. Early recognition and management are essential to optimize patient outcomes and mitigate complications.

## Introduction

Herpes zoster (HZ), commonly known as shingles, is a debilitating condition that typically affects adults and/or immunocompromised individuals and manifests as a painful, vesicular rash that follows a dermatomal distribution on the trunk, limbs, or face. This condition is caused by the reactivation of latent varicella-zoster virus (VZV) within the dorsal root ganglia of the spine, which can occur after a latency period of several years following a primary infection with VZV, commonly referred to as chickenpox.

HZ affects roughly one in three people in the United States during their lifetime [1]. The crude incidence of HZ in children under 18 years old was reported to be 74 per 100,000 person-years, 38 per 100,000 person-years among vaccinated children, and 170 per 100,000 person-years among unvaccinated children [2]. Routine varicella vaccination has significantly reduced the incidence of primary VZV infections and subsequent HZ cases in children. Nevertheless, children who have not been vaccinated or who have experienced natural varicella infection remain at risk for developing HZ. The diagnosis of HZ is usually made clinically, but it can be confirmed with polymerase chain reaction if deemed necessary [3]. Complications of HZ, such as secondary bacterial infections, post-herpetic neuralgia, and neurological sequelae, can significantly impact quality of life. Therefore, timely evaluation and management are important.

We report a case of a 10-year-old immunocompetent boy with a delayed vaccination schedule who developed HZ. This case emphasizes the critical role of routine childhood vaccinations in preventing primary infections and their associated complications. Additionally, it highlights the importance of considering HZ in the differential diagnosis of rashes in pediatric patients.

## Case presentation

A 10-year-old boy presented to the clinic, accompanied by his mother, for evaluation of a painful and itchy rash on the right side of his body. The mother reported that the boy initially developed gastrointestinal symptoms, consisting predominantly of vomiting, as part of a "stomach bug" that had affected nearly every member of their household. While other family members experienced similar gastrointestinal symptoms, this patient was the only one with a subjective fever measuring 99°F (37.2°C) during the illness.

Three days after his gastrointestinal symptoms began, the boy developed a rash that started along his right pectoralis major and gradually wrapped around to his mid-thoracic spine, following a T4-T5 dermatomal distribution. Five days after the onset of the rash, the mother brought the patient into the clinic for evaluation. The rash was intensely pruritic and associated with sharp, ice pick-like pain. At this visit, the child denied current systemic symptoms such as fever, chills, shortness of breath, nausea, vomiting, diarrhea, fatigue, or changes in appetite. No other family members had developed a rash, and no new foods, medications including antibiotics, detergents, sick contacts, and/or known environmental exposures were reported in his history.

The mother noted the child had a history of varicella infection at the age of two. His vaccination history was incomplete, as his immunizations were delayed. He received the diphtheria, tetanus, and acellular pertussis (DTaP), measles, mumps, and rubella (MMR), hepatitis A, and polio vaccinations late, between the ages of three and five years old, but never received the varicella vaccine. The mother tried treating the rash with over-the-counter cetirizine and hydrocortisone cream, which did not improve the rash or alleviate the pain or itching. Diphenhydramine was given by the mother at night, which mildly improved his ability to sleep.

**Figure 1:**
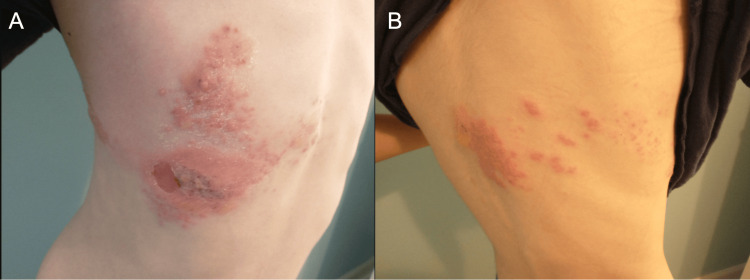
Clinical presentation of the patient’s vesicular rash on his chest (A) and back (B) that does not cross the midline.

On physical exam, the child appeared well-developed, well-nourished, and acutely uncomfortable due to the rash. Vital signs were within normal limits: blood pressure at 124/73 mmHg, pulse of 86 beats per minute, oxygen saturation at 98%, respiratory rate of 16 breaths per minute, and temperature of 97.5°F (36.4°C). Skin exam was significant for a right-sided, erythematous, vesicular rash in a T4-T5 dermatomal pattern that does not cross the midline anteriorly or posteriorly (Figure 1). Excoriations were noted along the anterior cluster of vesicles, which were also beginning to crust over. On a neurological exam, the patient had normal strength, tone, and reflexes, with no focal deficits. Cardiovascular, respiratory, musculoskeletal, and abdominal exams were unremarkable.

The clinical findings were consistent with HZ, commonly referred to as shingles, in the context of a previous varicella infection. The delayed presentation precluded the use of antiviral therapy (e.g., valacyclovir). Symptomatic management was continued, including cool compresses and steroids as needed for pain and pruritus. Follow-up was advised to monitor symptom resolution and ensure no complications developed. The mother reached out to the office a week later to provide an update that the patient’s rash was nearly resolved and no longer painful or pruritic.

## Discussion

Epidemiology and risk factors

The incidence of HZ has dramatically declined in the era of routine varicella vaccination, though similar cases have been reported in the literature [4]. Studies have demonstrated that children who have received the varicella vaccine have a 78% lower risk of developing HZ compared to their unvaccinated peers, with incidence declining further in those who received two doses [2]. Despite this, some vaccinated children may still develop HZ, with about half of these cases attributed to the vaccine strain and the other half to wild-type VZV [5]. Importantly, the vaccine strain exhibits lower neurovirulence, reducing the likelihood of severe complications, such as meningitis or encephalitis [6].

Children who experience natural varicella infection, particularly during infancy (less than one year old), are at heightened risk of HZ later in life [2]. This increased risk may be attributable to immature T-cell-mediated immunity during infancy, which could limit the effective containment of the virus in its latent phase. Additionally, children who develop a varicella-like rash following vaccination may have an increased likelihood of future HZ episodes [7].

Pathophysiology

HZ arises from the reactivation of latent VZV within the dorsal root ganglia, a process that may be initiated by immunosuppression or physiological stress. Unlike adults, where immunosenescence is the primary trigger, children may experience reactivation due to intercurrent infections or other stressors [5]. In this case, the patient’s history of natural varicella infection at age two, coupled with a recent viral illness, likely triggered HZ reactivation through an acute stress response.

Clinical presentation and differential diagnosis

The thoracic dermatomes are the most common site of HZ lesions in immunocompetent children, occurring here in 59% of cases [8]. In this patient, the characteristic vesicular rash in a unilateral dermatomal distribution and associated pain and pruritus facilitated the diagnosis. A detailed history and physical exam were essential in differentiating HZ from other differential diagnoses, including but not limited to contact dermatitis, insect bites, and other childhood exanthems.

Management and considerations

In most cases of pediatric HZ, antiviral therapy is unnecessary due to the typically mild disease course [9]. However, antiviral agents such as valacyclovir may be considered within 72 hours in severe cases or for immunocompromised patients to reduce viral replication and the risk of complications. Our patient presented beyond the optimal treatment window, so management was focused on providing symptomatic relief.

The most common complication of HZ in children is bacterial superinfection of the skin lesion, occurring in 5.2-9.3% of immunocompetent cases [10]. Serious complications, such as meningitis, sepsis, or facial palsy, are rare but more likely in immunocompromised individuals [10]. Disseminated HZ, reported in up to 40% of immunocompromised children, is exceedingly rare in healthy pediatric patients [11]. Post-herpetic neuralgia, a prevalent complication in adults, is uncommon in children but may cause significant morbidity in those with underlying immunodeficiencies [10].

Rare complications

Although rare, unique complications of HZ include HZ ophthalmicus, Ramsay Hunt syndrome, and laryngeal HZ. HZ ophthalmicus is HZ involvement of the ophthalmic branch of the trigeminal nerve that can result in facial rash, with up to half of these cases resulting in eye involvement and risk of vision loss [12]. This complication has been reported in vaccinated, immunocompetent children. Ramsay Hunt syndrome is HZ involvement of the facial nerve that presents with characteristic unilateral facial paralysis, otalgia, and vesicular lesions on the auricle or external auditory canal. In some cases, additional symptoms may arise from involvement of the vestibulocochlear nerve (e.g., hearing loss and vertigo) or the trigeminal nerve (e.g., trigeminal neuralgia) [12]. Laryngeal HZ is a rare manifestation of VZV reactivation that presents with severe throat pain, dysphagia, fever, and mucosal lesions visible on laryngoscopic examination. If left untreated, laryngeal HZ may be complicated by persistent cough and chronic throat discomfort, so it should be considered in the case of pharyngitis with poor response to antibiotics [13].

Clinical implications

This case highlights the critical role of varicella vaccination in reducing the risk and severity of HZ in children. While HZ is rare in this population, clinicians must recognize its potential occurrence, particularly in those with incomplete vaccination or a history of natural varicella infection. Pediatric HZ should remain on the differential diagnosis for vesicular rashes, and early recognition and appropriate management are essential to mitigate complications.

## Conclusions

HZ, though traditionally associated with older adults and immunocompromised individuals, can also occur in pediatric patients, particularly those with a history of natural varicella infection or incomplete varicella vaccination. This case underscores the importance of routine childhood vaccination in preventing primary varicella infections and reducing the risk of subsequent HZ reactivation. Although the incidence of pediatric HZ has declined significantly in the post-vaccine era, clinicians must remain aware of this condition when evaluating pediatric rashes, particularly in the context of recent illness or physiological stressors. Timely diagnosis, even in milder cases in healthy children, can help ensure appropriate management and prevention of complications. This case further highlights the role of vaccination as a critical tool not only in reducing the burden of varicella but also in mitigating the risks associated with HZ and its complications in pediatric populations.
